# A Dig into the Past Mitochondrial Diversity of Corsican Goats Reveals the Influence of Secular Herding Practices

**DOI:** 10.1371/journal.pone.0030272

**Published:** 2012-01-27

**Authors:** Sandrine Hughes, Helena Fernández, Thomas Cucchi, Marilyne Duffraisse, François Casabianca, Daniel Istria, François Pompanon, Jean-Denis Vigne, Catherine Hänni, Pierre Taberlet

**Affiliations:** 1 Paléogénomique et Evolution Moléculaire, Institut de Génomique Fonctionnelle de Lyon, Université de Lyon, Université Lyon 1, CNRS UMR 5242, INRA, Ecole Normale Supérieure de Lyon, 46 allée d'Italie, 69364 Lyon Cedex 07, France; 2 Laboratoire d'Ecologie Alpine, CNRS UMR 5553, Université Joseph Fourier, B.P. 53, 38041 Grenoble Cedex 9, France; 3 Centre National de la Recherche Scientifique, UMR 7209, Muséum National d'Histoire Naturelle, «Archéozoologie, Archéobotanique: Sociétés, Pratiques et Environnements», Département “Ecologie et Gestion de la Biodiversité” CP 56, 75005 Paris, France; 4 Department of Archaeology, University of Aberdeen, Aberdeen, United Kingdom; 5 Institut National de la Recherche Agronomique, UR 045 Laboratoire de Recherches sur le Développement de l'Elevage, Quartier Grossetti, 20250 Corte, France; 6 Laboratoire d'Archéologie Médiévale Méditerranéenne, CNRS UMR 6572, 5 rue du château de l'Horloge, BP 647, 13094 Aix-en-Provence, France; University of Florida, United States of America

## Abstract

The goat (*Capra hircus*) is one of the earliest domesticated species ca. 10,500 years ago in the Middle-East where its wild ancestor, the bezoar (*Capra aegagrus*), still occurs. During the Neolithic dispersal, the domestic goat was then introduced in Europe, including the main Mediterranean islands. Islands are interesting models as they maintain traces of ancient colonization, historical exchanges or of peculiar systems of husbandry. Here, we compare the mitochondrial genetic diversity of both medieval and extant goats in the Island of Corsica that presents an original and ancient model of breeding with free-ranging animals. We amplified a fragment of the Control Region for 21 medieval and 28 current goats. Most of them belonged to the A haplogroup, the most worldwide spread and frequent today, but the C haplogroup is also detected at low frequency in the current population. Present Corsican goats appeared more similar to medieval goats than to other European goat populations. Moreover, 16 out of the 26 haplotypes observed were endemic to Corsica and the inferred demographic history suggests that the population has remained constant since the Middle Ages. Implications of these results on management and conservation of endangered Corsican goats currently decimated by a disease are addressed.

## Introduction

In the Near East, cradle of the domestication process, goats were among the first to be domesticated ca. 10,500 years ago [1]–[4]. Several thousands years later, domestic goats (*Capra hircus*) were dispersed beyond the natural distribution of its wild ancestor (*Capra aegagrus*). They spread in Anatolia and Europe (starting from 8,800 calBP) throughout the Neolithic dispersal, along with pigs, cattle and sheep [5], [6]. Today, goats are present all over the world with more than 867 million of individuals [7]. In order to better assess the historical processes of domestication, goat mitochondrial genetic diversity has been largely studied across the old world (Europe, Asia, and more recently Africa). It is structured in six different haplogroups A, B, C, D, F and G [3], [8]–[16], with more than 90% of goats solely from the A haplogroup [14]. Moreover, a very weak phylogeographic structure is observed at the continent scale [8] contrary to other domestic species, such as sheep. Thus, A and C haplogroups have a worldwide distribution although B is mostly present in Asia. Some genetic structure is suspected however at a more restricted geographical scale [16] and some haplogroups, such as G and F, are now restricted to small regions (Middle-East [14] and Sicily [12] respectively).

If many studies have been dedicated to the characterization of the mainland diversity few were pursued on islands [12], [17]. However, large Mediterranean islands are of particular importance in the description of the genetic diversity of domestic species since they are considered as biodiversity hot spots, have a high degree of endemism and present a reservoir of cultural practices that have disappeared from the Mainland. In the case of goats, the study of genetic diversity on large Mediterranean islands is highly relevant for several reasons. First, goats are found on most of these islands from the beginning of the Neolithic diffusion and can serve as testimony of this spread [18]. Second, imported domestic goats, are physically isolated from their Mainland relatives. Third, breeding and husbandry practices on the Mediterranean islands are usually different to those on the continent because islands present large but restricted areas and preserve traditional practices mentioned previously. From these observations, we expect that mitochondrial diversity observed on islands can present evidence of historical events or ancient diffusion that would be lost elsewhere, such as the presence of the F haplogroup in Sicily that is unique outside the wild ancestor's area of distribution [3], [12], [14].

Here we characterize goat mitochondrial DNA (mtDNA) diversity through time by studying modern, but above all historical, goat populations from Corsica. We compared them with current continental or island breeds of the Mediterranean Basin to document the microevolution and the influence of insularity and husbandry practices on their genetic diversity. Corsica is an 183 km long and 83 km wide island located in the Northwestern part of the Mediterranean Basin. It formed a unique block with another island, Sardinia, during most of the Pleistocene until the land masses split approximately around 11,000 years ago. The presence of domestic Caprinae is attested in Corsica from the beginning of the Neolithic, ca. 7,600 calBP [19]. The herding system in Corsica, although close to other free-ranging and seasonal transhumance characteristic of other Mediterranean islands, displays a very interesting peculiarity namely the “wandering” of flocks in the mountains for weeks, between the end of the lactation period and the beginning of the births, under very loose surveillance from the herder [20]. These free-ranging practices in Corsica are not recent as Polybius had already mentioned them in his book XII during the II^nd^ century Before Christ. Nonetheless, being able to ascertain that transhumance and free ranging were common herding practices in Corsica since the Neolithic is a difficult task for zooarchaeologists [19]. Goats are known to be hardy, tough and able to adapt to very difficult habitats compared to other livestock. More than 200,000 goats were still present in Corsica less than 80 years ago but this number has decreased to 30,000 individuals during the last decades. Nevertheless, goats have retained a particular status in Corsica where pastoralism is still strongly established [21]. Besides, a Corsican breed has been recently recognized by the French CNAG (Commission Nationale d'Amélioration Génétique depending on Ministry of Agriculture) in 2003 and by decree of the French Ministry of Agriculture in 2007. It is a dairy breed, with relatively long hairs, either of uniform colour or multicoloured, characterized by its rustic character and ability to adapt to difficult grounds. This was possible because efforts were made to protect and promote local breeds by avoiding mixings with commercial breeds [22]. However, the traditional Corsican breed is now endangered because the traditional husbandry system seems difficult to maintain. The strains on this traditional system are not only economic but also social. Traditional goat husbandry practices implicate daily mobility from the herder, which most of the new generation does not wish to pursue [23]. More recently, the occurrence of Johne's disease (i.e., paratuberculosis) presents another very serious threat and has decimated flocks.

This study aims to (i) better characterize the current and past (medieval) mitochondrial genetic diversity of Corsican goats using a Control Region (CR) fragment; (ii) bring information about goat dispersal in the Neolithic by testing the congruence between scenario proposed and data observed on this island; (iii) explain the maintenance of endemic variability in Corsica; (iv) discuss implications for conservation of the Corsican breed. For these purposes, we gathered samples of 28 present-day individuals and 29 bones dated from the Middle Ages (XII^th^ and XIV^th^ centuries).

## Methods

### The archeological site of Rostino

**Figure 1:**
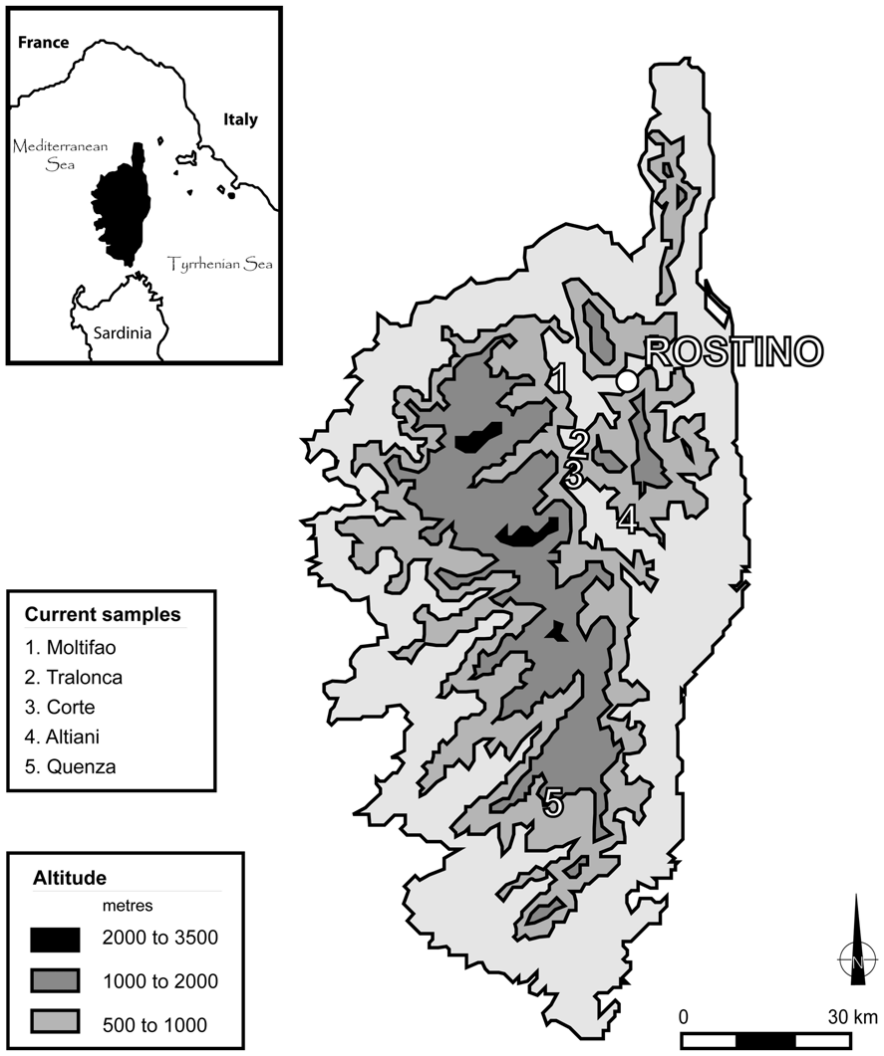
Ancient and present-day sampling of goats in Corsica. The medieval samples are all located in the archeological site of Rostino, in the northern part of the island. The present-day samples come from 5 different localities identified by numbers on the map.

The castrum (strong hold) of Rostino [24] is situated in the North East of Corsica (Figure 1). Occupied between the XII^th^ and XIV^th^ centuries AD, this late medieval site has yielded the largest assemblage of Caprinae in Corsica in a good state of preservation [25], which is rare in the acidic soil of Corsica [19]. Among the domestic species of the XIV^th^ century deposit, Caprinae represent more than 70% of the identified mammal remains [25]. The economy of the castrum relies on specialized caprine exploitation where the production of sheep and goat complement each other: sheep for meat production and goat for milk and hair production [25]. During the XIV^th^ century, the caprine exploitation specialized in goats with *Capra hircus* representing more than 70% of the total caprine remains [25]. This large amount of late medieval Corsican *Capra hircus* represents a great opportunity to investigate the consequence of the secular herding practices and the selective choices made by herders to renew their flock given the genetic diversity of goats in large Mediterranean islands.

### Archeological samples

We analysed 29 bone fragments from Rostino: 17 were excavated from a XII^th^ century deposit and 12 from a deposit dated to the XIV^th^ century. These bones have been identified as *Capra hircus* using morphoscopic criteria on both dental [26], [27] and appendicular [28]–[30] characters (Table S1). There is no ambiguity about the origin of bones from domestic animals as the wild ancestor (*Capra aegagrus*) has never been present in Europe [31]. According to the type of bone (mandible, radius, humerus …), the laterality (left, right) and detailed information from the excavation, we were able to clearly identify 25 different individuals from the 29 fragments. The four other fragments very probably came from previously identified individuals (Table S1). Two samples were subsequently identified as sheep by the molecular analyses. This is not surprising as the inter-specific distinction between sheep and goat on fragmented bones cannot be ascertained with a 100% reliability [29]. Molecular analyses have precisely proven to be useful in this case [32] as has, more recently, the analysis of collagen by mass spectrometry [33].

### Ancient DNA extracts and PCR amplifications

Retrieval of the DNA preserved in bones was performed in ultra clean rooms dedicated to ancient DNA experiments (French National Platform of Paleogenetics PALGENE, CNRS, ENS Lyon). No more than 4 *capra* samples were treated in the same session of DNA extraction along with a bone from another species (cervids, ursids) and a blank control [34], [35]. 100 to 500 milligrams of each bone fragments were reduced in powder and suspended in 5 or 10 ml of EDTA buffer as described in [36]. We extracted the DNA using one, or both, of the two following protocols: a classical phenol-chloroform approach [36], [37] or direct purification using Qiaquick column (Qiagen kit) [34]. 25 out of the 29 samples were independently re-extracted in a second laboratory dedicated to ancient DNA in another city, Grenoble, using the Qiaquick protocol.

A 130 bp fragment of the CR (HVI) was amplified with the CapFII and CapRII primers with conditions identical to those described in [38]. At least 2 or 3 independent positive amplifications per sample were obtained, cloned and sequenced following protocols described in [34], [35]. The final sequence of one individual was obtained by making the majority-rule consensus of all consensus of all different clones obtained from each of the independent amplification. More than 80% of the differences observed between clones were G to A or C to T punctual substitutions. This result is consistent with ancient DNA degradation profiles where deamination of cytosines is known as the major factor of artifactual substitutions [39].

### Present-day Corsican goat sequences

We sampled 28 goats in 5 different localities in Corsica (7 from Moltifao, 6 from Tralonca, 11 from Corte, 3 from Altiani, 1 from Quenza; Figure 1, Table S1). 14 sequences were already published [14] and 14 were produced for this study. To amplify the CR fragment we used the same primers (CapFII and CapRII) than for medieval sequences with slightly modified conditions: 40 cycles were performed instead of 50–60 for ancient DNA and products were directly sequenced on both DNA strands.

### Sequence analyses

All the sequences obtained were aligned (Seaview v4 [40]) and the primers removed leading to a fragment of 130 base pairs. Four data sets were constituted: (i) all medieval sequences, (ii) the XII^th^ century sequences, (iii) the XIV^th^ century sequences and (iv) the present-day sequences.

Firstly, the mitochondrial haplogroup of each Corsican sequence found was determined by performing phylogenetic analyses. The different Corsican haplotypes were analyzed together with 20 haplotypes of known haplogroups. These 20 haplotypes corresponded to the 20 reference sequences that were different for the 130 bp fragment under study, in the dataset selected by Naderi et al. [14] to represent the worldwide variability of the whole HVI-control region (558 bp). Identical sequences, or haplotypes, were identified in the Corsican dataset by using Fabox [41]. After estimating the better model of evolution using jModeltest program [42] and the Akaike Information Criterion (AIC), we performed Bayesian analyses (BA) with MrBayes v3.1.2 [43], [44] (independently confirmed by Maximum Likelihood analyses, not shown). The parameters used for BA were the following: GTR+I+G (nst = 6 and rates = invgamma), 5,000,000 generations sampled every 1000^th^ generation, 4 chains, a burn-in period of 500 trees (i.e. 10% of generations) visually confirmed using Tracer v1.4.1 (developed by Rambaut A and Drummond A; available from http://beast.bio.ed.ac.uk/Tracer), allcompat option. Two independent runs were performed with an average standard deviation of split frequencies at completion of 0.006598. The average values obtained for alpha and proportion of invariable sites parameters were 0.287 and 0.323 respectively.

Secondly, we assessed the relationships between only medieval or all Corsican sequences, by performing median-joining networks, using the Network software ([45], available at fluxus-engineering.com) with default parameters. Network Publisher was used to manipulate the networks. To compare the Corsican mitochondrial genetic diversity with the Mediterranean or worldwide diversity, we defined supplementary datasets, one by haplogroup observed, gathering all the sequences published and covering the 130 bp fragment. These datasets combined with the Corsican sequences were used to draw median-joining networks (references are given in the legends of Figures S1 and S2).

Thirdly, to assess the past demographic history of the Corsican goats we performed a Bayesian Skyline Plot (BSP) using BEAST v.1.5.4 software [46]. All the Corsican sequences were used and average tip dates were given for all medieval sequences: 850 years BP for the XII^th^ century and 650 years BP for those of the XIV^th^ century. BEAUti v1.5.3. was used to build the xml file by using the following parameters: HKY+I+G4 (best model for this dataset assessed by jModeltest and AIC criteria [42]); uncorrelated lognormal relaxed clock; 5 groups and 100 millions of iterations with parameters saved every 10 000 iterations; Burn-in: the first 10% were discarded. The results of 4 independent runs were analysed and the Bayesian Skyline Plot reconstructed with Tracer v.1.4.1 (Figure S3).

Finally, to describe the genetic diversity observed for the Corsican goats, we computed different classical parameters using either DnaSP v5 [47] or Arlequin v3.5.1.2 [48] including haplotype diversity and frequencies, sequence diversity, pairwise comparisons, and population comparisons (*F_ST_*, Fu tests). We compared the different Corsican datasets with each other but also with other datasets corresponding to mainland or island populations (see [16] for accession numbers and geographical origin). We focused in particular on Sardinia's island (75 sequences, accession numbers FJ571522 to FJ571596, [17]; Corsica's closest island) and Portugal (the biggest dataset generated for mainland goats, 288 sequences, accession numbers AY961629 to AY961916, [11]). We also considered 4 different datasets (see Supporting Information for details and references) corresponding to the southern or the northern area of the Mediterranean Sea, the Mediterranean islands (Sicily and Sardinia), and finally 8 Neolithic goats of Baume d'Oullens [38]. To reduce possible biases due to large differences in the size of the datasets (low number of Corsican sequences), we randomly sampled 49 sequences of the non-Corsican sequences and repeated this operation at least three times. The genetic parameters estimated by DnaSP v5 [47] or Arlequin v3.5.1.2 [48] were then computed on these resampled datasets of equal size.

## Results

### Medieval goat samples

**Figure 2:**
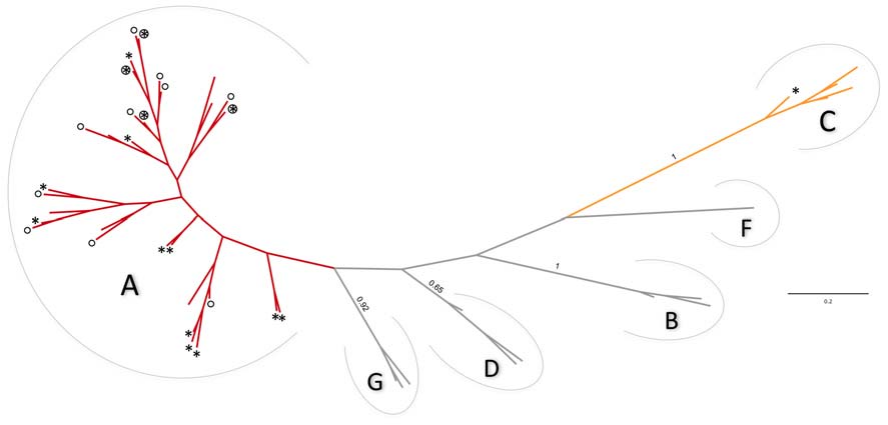
Mitochondrial diversity of Corsican goats compared to world-wide goats diversity. Ancient (circles) and current (stars) Corsican sequences have been used with 20 sequences of reference corresponding to all haplogroups described up to now [14] to reconstruct a Bayesian phylogenetic tree using a GTR+I+G model of evolution (see text). Only a single sequence by haplotype has been used, so haplotypes in common between medieval and today periods are indicated by both a circle and star. Only posterior probabilities higher than 0.6 are indicated.

**Figure 3:**
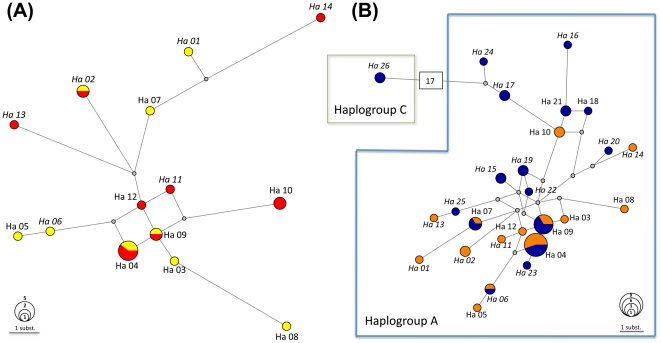
Networks generated with CR sequences of Corsican goats from A) medieval samples only or B) combined with present-day samples. The circles are proportional to the number of sequences obtained and colours indicated the period: yellow corresponds to XII^th^ century, red to XIV^th^ century, orange to all medieval and blue to present-day sequences. See Table 1 for details about haplotypes and archeological samples. Haplotypes indicated in italics are those only observed in Corsica up to now. Haplotype Ha 26 is the only one from the haplogroup C and differs from the closer haplotype by 17 positions that is materialized by a square with this number.

We analyzed 29 bones and obtained reproducible and congruent sequences of *Capra hircus* for 25 of them (Table S1). We are confident that these results are authentic as we obtained the same results in both laboratories. We also took the ancient DNA precautions recommended by the community as we are used to do (e.g. [34], [35], [38], [49], [50]). When different bones were supposed to be from a single individual (Ro-5, Ro-10 and Ro-22), systematically we obtained the same sequence confirming the first assessments. Finally, we determined 21 sequences coming from different individuals: 10 dated to the XII^th^ century and 11 dated to the first half of the XIV^th^ century. All medieval sequences were from the A haplogroup as shown by the phylogenetic analysis (Figure 2). The six haplogroups appeared monophyletic and were supported by posterior probabilities (pp) higher than 0.9 for 3 of them B, C, G (F is not concerned as only one sequence is used). The A haplogroup that had the highest number of sequences and that was the more diverse received the lowest support (pp<0.5). Nevertheless, the clustering of the newly determined sequences inside the A haplogroup raises no doubt as confirmed by subsequent network analyses (see also mismatch distributions, Figure S4). Substantial diversity is observed among medieval haplotypes as seen on the network (Figure 3A). Among the 21 sequences, we detected 14 unique haplotypes (Table S1) with haplotype Ha 04 being the most frequent (5 individuals). Six out of the 14 haplotypes have never been described before. On average, the mean number of pairwise differences observed between sequences reaches 4.65±2.37 (Table 1, Figure S4). The diversity appeared not significantly different between the XII^th^ century with 9 haplotypes for 10 sequences (13 polymorphic sites) and the XIV^th^ century with 8 haplotypes for 11 sequences (15 polymorphic sites). According to the network performed on all medieval sequences (14 haplotypes, 19 polymorphic sites), only 3 haplotypes were shared between both periods (Figure 3A). Two of them had a central position in the network (Ha 04 and 09; Figure 3A).

**Table 1:** Genetic diversity parameters.

	Number of sequences	Number of haplotypes	Haplotype diversity	Mean number of pairwise differences	Nucleotide diversity	Fs (Fu)	Tajima D
**Corsican goats**							
XII^th^ century	10	9	0.9778±0.0540	4.82±2.57	0.0371±0.0224	−3.7113 (p: 0.0200)	0.2247 (p: 0.6240)
XIV^th^ century	11	8	0.9273±0.0665	4.62±2.45	0.0355±0.0213	−1.6798 (p: 0.1530)	−0.4355 (p: 0.3440)
All Medieval	21	14	0.9381±0.0388	4.65±2.37	0.0358±0.0204	−5.0723 (p: 0.0130)	−0.4484 (p: 0.3590)
Present Corsican	28	16	0.9524±0.0208	7.01±3.40	0.0540±0.0291	−2.8511 (p: 0.1280)	−0.2150 (p: 0.4590)
Present Corsicanhaplogroup A only	26	15	0.9477±0.0238	4.90±2.47	0.0377±0.0211	−4.3581 (p: 0.0380)	−0.0555 (p: 0.5510)
All Corsican (Med+Pre.)	49	26	0.9473±0.0189	6.12±2.96	0.0471±0.0253	−9.8130 (p: 0.0010)	−0.3022 (p: 0.4350)
All Corsican (Med+Pre.)haplogroup A only	47	25	0.9436±0.0204	4.85±2.41	0.0373±0.0206	−11.9233 (p: 0.0000)	−0.0826 (p: 0.5300)
**Other goats**							
Present Sardinian	75	46	0.9827±0.0052	5.68±2.75	0.0437±0.0235	−25.3300 (p: 0.0000)	−0.7342 (p: 0.2470)
Present Portuguese	288	104	0.9790±0.0030	5.30±2.60	0.0414±0.0221	−24.8789 (p: 0.0000)	−0.7741 (p: 0.2560)

The different parameters for all datasets (see text) have been computed on a 130 bp fragment of the HVI region of the mitochondrial control region (CR). The values are given with their confidence interval (±) and tests with their p-value (p).

### Present-day goat samples

28 individuals from 5 different localities were studied (Table S1 for details). Two different mitochondrial haplogroups were observed (A and C; Figure 2) with a higher proportion of A sequences (92.8% i.e. 26 out of 28 sequences). Considering all A and C sequences, the mean number of pairwise differences reaches 7.01±3.39 (29 polymorphic sites; Figure S4). However, when only sequences of the A haplogroup were considered, this value drops to 4.90±2.47 (19 polymorphic sites, Table 1) which is close to the one observed for A medieval goats. The two sequences from the C haplogroup came from the same locality, Tralonca (Figure 1), and shared the same haplotype Ha 26 that has not been described elsewhere (Figure 3B). 15 haplotypes were obtained for the 26 sequences of the A haplogroup among which, 10 were only observed in Corsica. The most frequent haplotypes were, like for the late medieval goats, Ha 04 and Ha 09, with 4 individuals from 3 different localities in both cases (Figure 3B).

### Comparison of Corsican goat diversity through time

No significant difference was observed between the goats of the XII^th^ and the XIV^th^ centuries (non significant *F_ST_-*value; Table S2). The difference became significant when medieval and present goats were compared (0.036, p-value 0.027). This was due to the presence of the C haplogroup in the present-day sequences since the test was no more significant when only A sequences were taken into account (0.027, p-value 0.099). Finally, there were no significant differences between the medieval and present-day sequences, with or without considering the C sequences. Medieval and present-day goats shared 4 haplotypes (Ha 04, 06, 07 and 09, Figure 3B) but only one was specific to Corsica (Ha 06). The 3 remaining haplotypes were generally frequent in other populations.

No significant changes were observed in the demographic history of the Corsican population using the Bayesian Skyline Plot (Figure S3). Neither a sign of expansion nor of a crash were observed from the medieval times to date, as rather a constant population size pattern was obtained. This is congruent with the Tajima's D values obtained (Table 1) but not with all the Fu's Fs values computed. However, this latter parameter can reflect other factors than population growth (selection, bottleneck, … [16]).

### Comparison of Corsican goat diversity with other geographical places

We compared the diversity observed in Corsica with other Mediterranean island populations and mainland breeds. The 75 sequences of Sardinia were clustered in 46 haplotypes that were all from the A haplogroup [17]. Similarly, the 288 Portuguese sequences were represented by 104 haplotypes, from which only one was from the C haplogroup and all the others from the A haplogroup [11]. By expanding the comparison to larger or other areas, we observed that the C haplogroup is described only in Europe (in present-day Northern Mediterranean area and already at the Neolithic time, Table S3 and not shown). Both the medieval and present-day Corsican groups appeared significantly different from all other groups whatever the resampled datasets taken into account (Table 1, Tables S2 and S3). Similarly, all the non-Corsican groups of goats also appeared significantly different from each other (data not shown). However, four different haplotypes (Ha 05, 08, 09 and 10) were shared between Corsica and Sardinia islands and six (Ha 07, 08, 09, 10, 12 and 21) between Corsican and Portuguese goats.

The median-joining network performed on 39 worldwide sequences of the C haplogroup (Figure S1) revealed a classical expansion structure with European sequences on one side and Asian sequences on the other (with the single exception of one Swiss haplotype; [8]). As expected, the sequences obtained from Neolithic goats of the archeological site of Baume d'Oullens in France [38], among the first goats to have been diffused in Europe, appeared in the central part of the European cluster. The Corsican haplotype showed the highest number of substitutions with this central node (3 substitutions). The same analysis performed on the sequences from the A haplogroup restricted to the Mediterranean area, showed a more complex history with no clear emerging pattern (Figure S2).

## Discussion

### DNA preservation in medieval samples

From the 29 archeological bones dated back to the XII^th^ and XIV^th^ centuries, we obtained 21 sequences from different ancient goat individuals: 10 from the XII^th^ century deposit and 11 from the XIV^th^ century deposit, which represents a surprisingly high DNA preservation for remains in Corsica. Such a good preservation of the DNA, which is here correlated to the good preservation of the bones themselves like in most late Medieval Corsican sites [19], [51], is probably due to the recent age of the site.

### Characterization through time of mitochondrial genetic diversity in Corsican goats

The comparison of Middle Ages and present mitochondrial diversity was carried out using 28 present-day goats from five different localities. This comparison may be slightly biased by differences existing in the time span and geographic distribution for either ancient or modern samples. Indeed, the sampled geographic area is larger for the modern goats (Figure 1) whereas the time span is longer for the ancient samples.

Our results tend to prove that the mitochondrial diversity of Corsican goats has remained relatively constant since the Middle Ages. Moreover, we detect no significant demographic changes (*F_ST_*, BSP and Table 1) or decrease of genetic diversity. The only difference between both periods is the occurrence of two C haplotypes in the present-day samples, all other goats belonging to the A haplogroup. However, given a dataset of 21 medieval individuals and assuming a constant frequency of the C haplogroup in Corsica (2/28 = 0.0714), we have a 21% of chance of having missed this haplogroup in the Middle Ages sampling.

### The occurrence of the C haplogroup in Corsican goats in the context of the Neolithic diffusion

The presence of the A and C haplogroups in Corsica is in agreement with the goat mitochondrial variability observed in European countries. Most of European goats are from the A haplogroup, with C haplotypes found at a rare frequency in Switzerland, Portugal, Spain and Slovenia [3], [8], [11], [14]. This is also consistent with previous paleogenetic studies that already detected both haplogroups in goats from Southern France in the early Neolithic period [38]. The median-joining network performed on all the C haplotypes found worldwide (Figure S1) gave results congruent with the diffusion of goats in the Neolithic. Indeed we observed: i) a clear separation of the European haplotypes from the Asian ones with only one exception, a shared haplotype between China and Switzerland; ii) a star-like pattern for European haplotypes suggesting a population expansion with a central position for the Neolithic haplotypes [38]; iii) a divergent haplotype for Corsica compatible with a subsequent isolation.

Without a doubt, the origin of the C haplogroup in Europe can be traced back to the Neolithic spread where its frequency was probably higher than the one observed now [38]. Today, the C haplotypes are relatively scarce in goats and appear more like reminiscent testimonies of this first diffusion. Interestingly, not one has been detected yet in the Southern Mediterranean area or in other Mediterranean islands than Corsica (see Supporting Information). According to the few data we have, it is difficult to conclude on the ancient origin of the Corsican C haplotype or a more recent origin linked to subsequent exchanges with the Northern Mediterranean mainland. However, its position in the network is compatible with the first explanation. Further ancient DNA studies, for a larger area and older period, would be very interesting to highlight this question.

### Variability in Corsica vs other Mediterranean areas

The relative stability through time of goat diversity in Corsica could be explained by regular importation of goats from other continental areas or islands, e.g. for commercial trade, as many contacts by sea have been reported during the last centuries in the Mediterranean area [52]. However, a striking point is that the diversity observed in Corsica is substantial for both periods (Middle Ages and present-day, Figure 3A and Figure 3B respectively) and differs from that of other places (Table 1, data not shown); only about half of the Corsican haplotypes (14/26) are shared with goats from other geographical regions despite our study focused on a short CR fragment (130 bp). This result is not unexpected however. Previous studies - usually targeting the 480 bp fragment first described in [8] and covering our shorter fragment - have already shown that goats were more polymorphic than other livestock (cattle, sheep, pig) on the CR [11], [15], [53]. Moreover, the specific analysis of the A haplogroup (more than 90% of the modern goats, [14]) confirmed high haplotype diversities for 20 populations/countries with values close to one [16]. Pereira et al. [16] showed a strong correspondence between mitochondrial genetic and geographic distances suggesting that after the initial expansion, differentiation among regions has been established and maintained [16]. A similar conclusion was obtained with large-scale nuclear SNP analyses obtained for 16 breeds of goats [54] and from microsatellites analyses including Corsican and other European goat populations [55]. This seems in agreement with what we observed in Corsica when compared with other Mediterranean populations (*F_ST_* comparisons, Table S2, data not shown).

### Implication of the traditional Corsican husbandry in the maintenance of the variability

Except for some haplotypes that are common in many countries (Figure S2) and probably constitute traces of the initial diffusion, 46% of the Corsican haplotypes found have not been previously described elsewhere. The preservation through time of this endemic genetic diversity and its constant level since medieval time could suggest that relatively large effective population sizes have been maintained in domestic goats through exchanges of animals. But ethnographic insights into the herding practices carried out in the Niolu [20] provide another possible or complementary explanation for the preserved genetic diversity of the Corsican goat breed.

Typically in the past for the Mediterranean area, goats were usually moved according to the seasonal changes (transhumance) to gain access to more reliable food. Because fodder resources fluctuate in the wild according to different factors (e.g. annual climatic conditions), the system developed in Corsica has been extensive with goats left free-ranging most of the times although under the careful control of the herders. This particular system led to the characteristics observed in the Corsican goats [21], [56]: i) the high diversity of coat colours is encouraged as individuals can be more easily identified by sight; ii) only the strongest and toughest goats can generally survive in this relatively hostile environment, explaining why the introduction of goats from industrial breeds usually failed; iii) large herds are usually managed to maximize the herd's productivity overall instead of individual productivity. Indeed, selection is not performed to optimize for instance, either milk productivity or the fecundity, but instead to obtain a constant productivity of the herd by year whatever the weather or difficulties encountered [57]. All these points lead to a strategy where the phenotypic diversity, and the underlying genetic diversity, is maximized in order to obtain herds that are highly adaptable, rustic and robust.

In order to do that, herders exert strong selection pressure while forming their herds to gain in productivity but also to favour behavioural traits. Indeed, along with the search for constant productivity, one of the main objectives of herders is to design flocks that will maintain “families” from the same maternal lineage [20]. A young descendant female is usually chosen according to its mother's and grandmother's family, taking into account its desirable productive traits as well as its abilities to endure the tough conditions. Animals coming from the same family/pool will live together and move together more easily and naturally. Such “familial” behaviour is extremely beneficial to the cohesion of herds under free-ranging exploitation and especially during the movements on the pasturelands (transhumance). On the contrary, introduction of new animals from other herds can induce significant disturbance in the herd movements by breaking the cohesion of the group. This practice of herders of course has the effect of reducing the genetic diversity. Hence, to insure the persistence of the genetic diversity and to “change the blood”, “cambià u sangui” in the language of traditional Corsican herders, selected males of these different maternal lineages or “families” will be exchanged between flocks to avoid inbreeding and, without control, renew the pool of the mating males within a seasonal time span. As mitochondria are inherited maternally, this system will naturally lead to maintaining high mitochondrial genetic diversity between herds or “families”. So, the diversity we observed would be not promoted by large herds with many exchanges of females between them but on the contrary by traditional practices. These ones rely on very few introductions of females coming from other herds to keep the kinship within the herd as tight as possible to reinforce cohesive behaviour during the ranging but also on a mixing of the genetic diversity through the exchanges of males only.

Such entangled practices in the Corsican husbandry system can explain why we do not observe change of the mitochondrial diversity in Corsican goats since the late Medieval period, and probably earlier if we had been able to investigate the genetic diversity of goats from earlier periods. It's thus highly probable that sustainable husbandry practices in today's Corsica that are so well adapted to their environment are, at least partly, the result of practices over millenaries contributing to the maintenance of a relatively high level of genetic diversity. Finally, analyses of additional samples and genetic markers coupled with population simulations using varying population genetic parameters should help to test between both hypotheses, regular importation of goats or millenaries husbandry practices.

### Towards a protection of the Corsican goat breed

In this study, we observed that the mitochondrial diversity of goats in Corsica Island has been maintained since the Middle Ages to date. In a time where rustic breeds are endangered and industrial breeds tend to reduce the genetic resources [22], Corsican goats, by the model of husbandry used and the high diversity observed constitute an interesting pool to preserve for the future management of domestic genetic resources. All the actors concerned by the Corsican goat (regional association in charge of its management, public authorities, researchers and extension services) should so pay attention to make their preservation successful all the more because paratuberculosis has started to decimate flocks.

### Ethic statement

The medieval bones of goats were excavated from the Rostino archeological site. Daniel Istria, in charge of the excavation, authorized their analyses. Tissue samples were collected in Corsica in the framework of the ECONOGEN project (European Union contract QLK5-CT-2001-02461), following the European ethical rules implemented in all European projects.

## Supporting Information

Figure S1 **Network generated with CR sequences of the C haplogroup (130 bp).** Only domestic goats have been considered (35 sequences; Luikart et al. 2001 [8], Sultana et al. 2003, Joshi et al. 2004, Pereira et al. 2005 [11], Chen et al. 2005, Liu et al. unpublished, Naderi et al. 2007 [14]). Neolithic sequences coming from the archeological site of Baume d'Oullen (Fernández et al. 2006 [38]) were also taking into account (4 sequences).
(DOC)

Figure S2 **Network generated with CR sequences of the A haplogroup (130 bp) for goats coming from the Mediterranean Sea around Corsica.** 584 sequences were used for the median-joining network analysis coming from: North Mediterranean area (Italy, France, Spain), Mediterranean Islands (Malta, Sicily, Sardinia), South Mediterranean area (Morocco, Algeria, Tunisia). See Pereira et al. 2009 [16] for the accession numbers of the sequences used and their geographical origin. Positions were weighted inversely to the number of mutations observed by position on a first run-test.
(DOC)

Figure S3 **Bayesian Skyline Plot.** The analyses were performed using the 49 Corsican sequences with date-tips for the medieval sequences (see text for details). X-axis: Time in years; Y-axis: Population size (Neτ) in log-scale. Mean is plotted with the 95% HPD. 3 runs of 100 M of iterations were performed (ESS >200 for all parameters).
(DOC)

Figure S4 **Mismatch distributions for medieval and extant Corsican goat populations.** The numbers of pairwise differences are given on the x-axis and their frequency on the y-axis.
(PDF)

Table S1 **Corsican goats sampling and mitochondrial genotyping results.** A star indicates archeological samples for which the molecular identification was *Ovis aries* and a dash when no amplification was obtained.
(DOC)

Table S2 Population pairwise *F_ST_*. Pairwise difference was used as distance method. *F_ST_* values are given at upper right corner and corresponding p-values at bottom left. Significant p-values are highlighted in color (orange for intra-Corsica comparisons, pink for inter comparisons). NS: Non-significant, * p-value between 0.05 and 0.01, ** p-value between 0.01 and 0.001, *** p-value < to 0.001.
(DOC)

Table S3 Genetic diversity parameters. Comparison for A and C haplogroups between Corsican and other Mediterranean or Portuguese datasets (see Figures S1, S3 and text).
(DOC)
