# Supplementary material for: A Dig into the Past Mitochondrial Diversity of Corsican Goats Reveals the Influence of Secular Herding Practices
Source: PLoS One. 2012 Jan 27;7(1):e30272. doi: 10.1371/journal.pone.0030272 (PMC3267719; doi:10.1371/journal.pone.0030272)
Supplement: Figure S1 — Network generated with CR sequences of the C haplogroup (130 bp). Only domestic goats have been considered (35 sequences; Luikart et al. 2001 [8], Sultana et al. 2003, Joshi et al. 2004, Pereira et al. 2005 [11], Chen et al. 2005, Liu et al. unpublished, Naderi et al. 2007 [14]). Neolithic sequences coming from the archeological site of Baume d'Oullen (Fernández et al. 2006 [38]) were also taking into account (4 sequences). (DOC) [file pone.0030272.s001.doc]

**
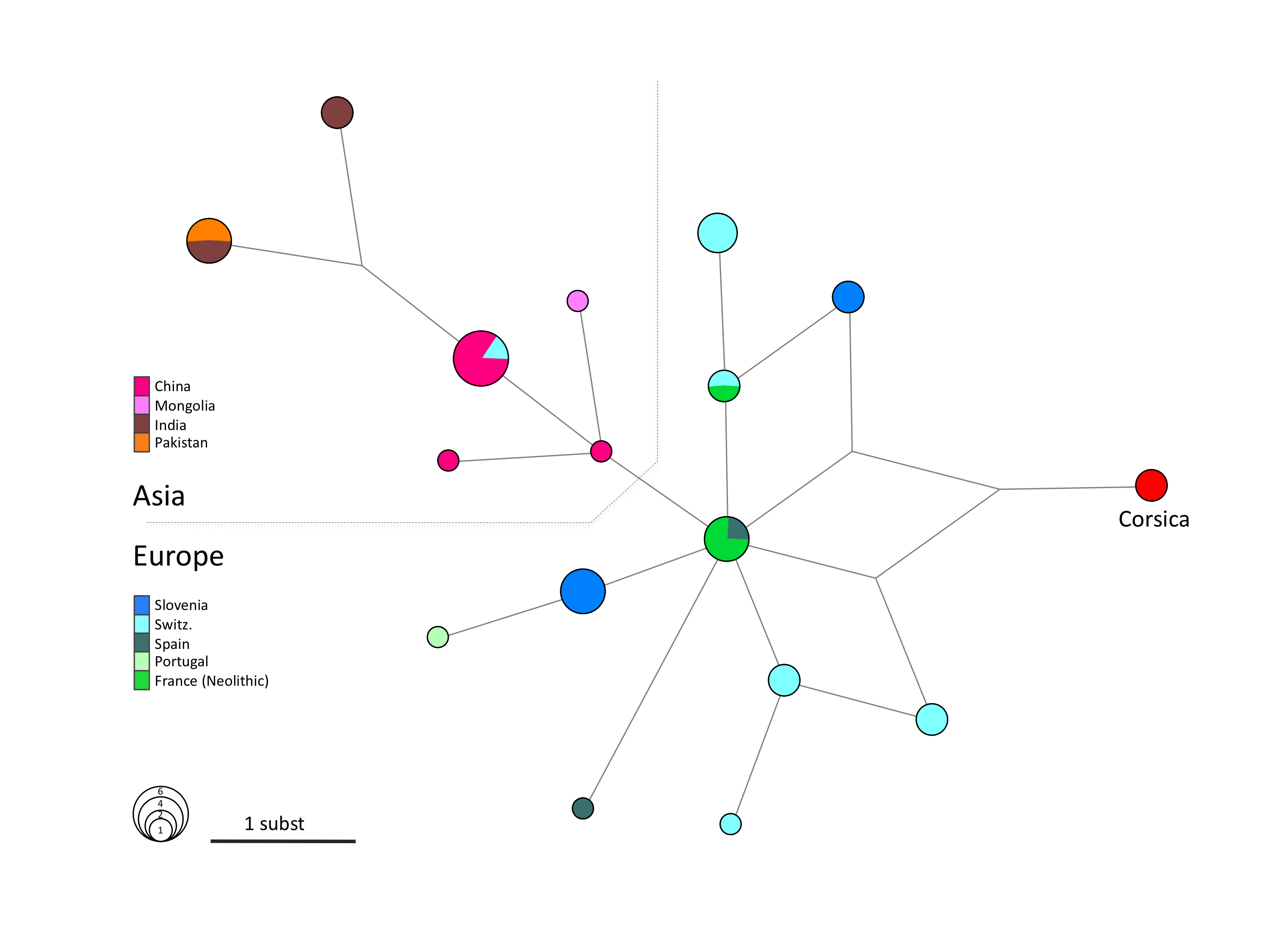
**

**Supplementary references:**

Sultana, S., Mannen, H. & Tsuji, S. 2003 Mitochondrial DNA diversity of Pakistani goats. *Anim. Genet.* **34** (6), 417-421

Joshi, M.B., Rout, P.K., Mandal, A.K., Tyler-Smith, C., Singh, L. & Thangaraj, K. 2004 Phylogeography and origin of Indian domestic goats *Mol. Biol. Evol*. **21** (3), 454-462

Chen, S.Y., Su, Y.H., Wu, S.F., Sha, T. & Zhang, Y.P. 2005 Mitochondrial diversity and phylogeographic structure of Chinese domestic goats. *Mol. Phylogenet. Evol.* **37** (3), 804-814

Liu, R.-Y., Lei, C.-Z. & Yang, G.-S. 2006 Mitochondrial DNA diversity of Chinese goats. Unpublished
