# Supplementary figures and images for: A Dig into the Past Mitochondrial Diversity of Corsican Goats Reveals the Influence of Secular Herding Practices
Source: PLoS One. 2012 Jan 27;7(1):e30272. doi: 10.1371/journal.pone.0030272 (PMC3267719; doi:10.1371/journal.pone.0030272)

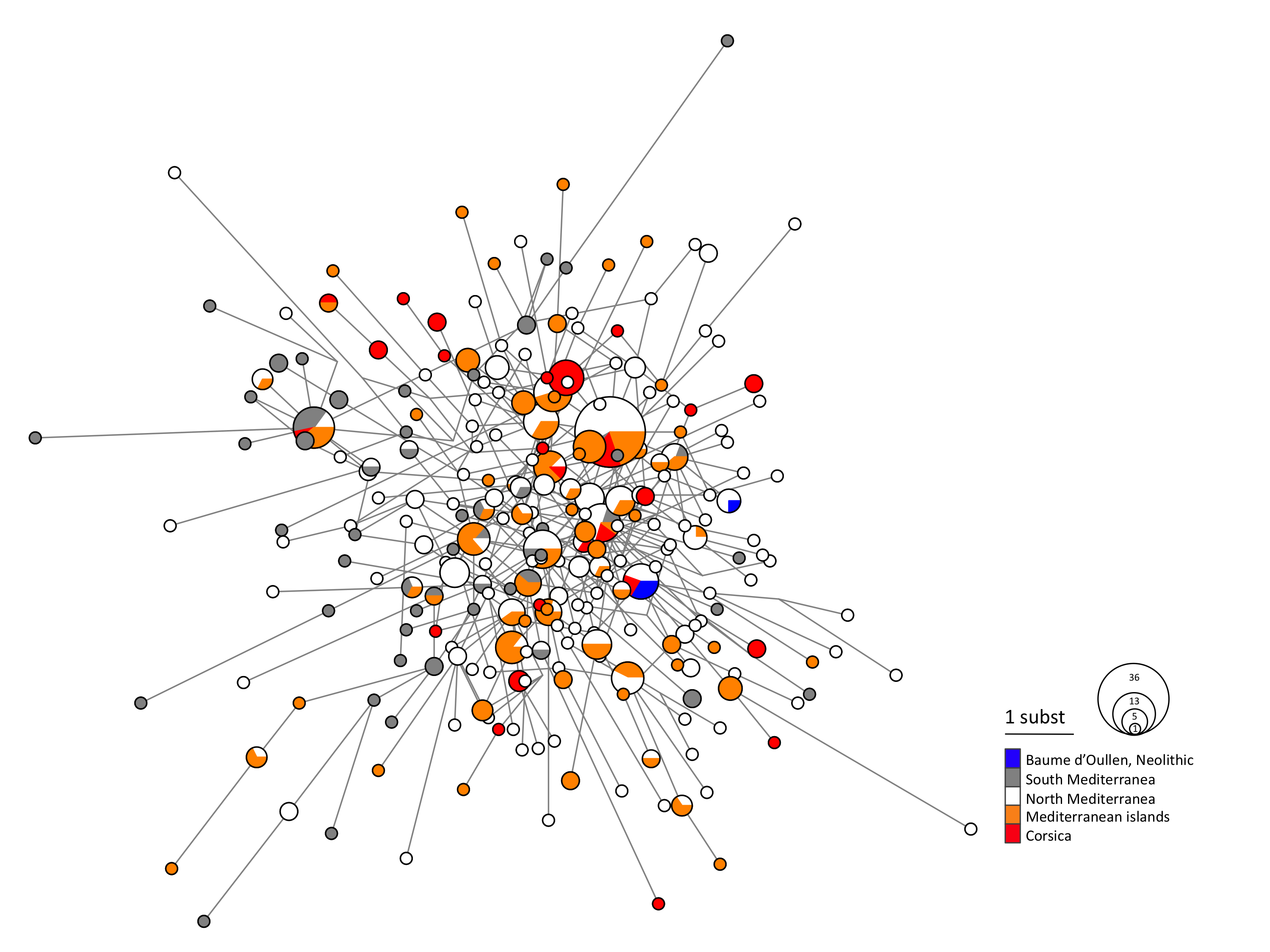

Supplement: Figure S2 — Network generated with CR sequences of the A haplogroup (130 bp) for goats coming from the Mediterranean Sea around Corsica. 584 sequences were used for the median-joining network analysis coming from: North Mediterranean area (Italy, France, Spain), Mediterranean Islands (Malta, Sicily, Sardinia), South Mediterranean area (Morocco, Algeria, Tunisia). See Pereira et al. 2009 [16] for the accession numbers of the sequences used and their geographical origin. Positions were weighted inversely to the number of mutations observed by position on a first run-test. (DOC) [file pone.0030272.s002.doc]

**
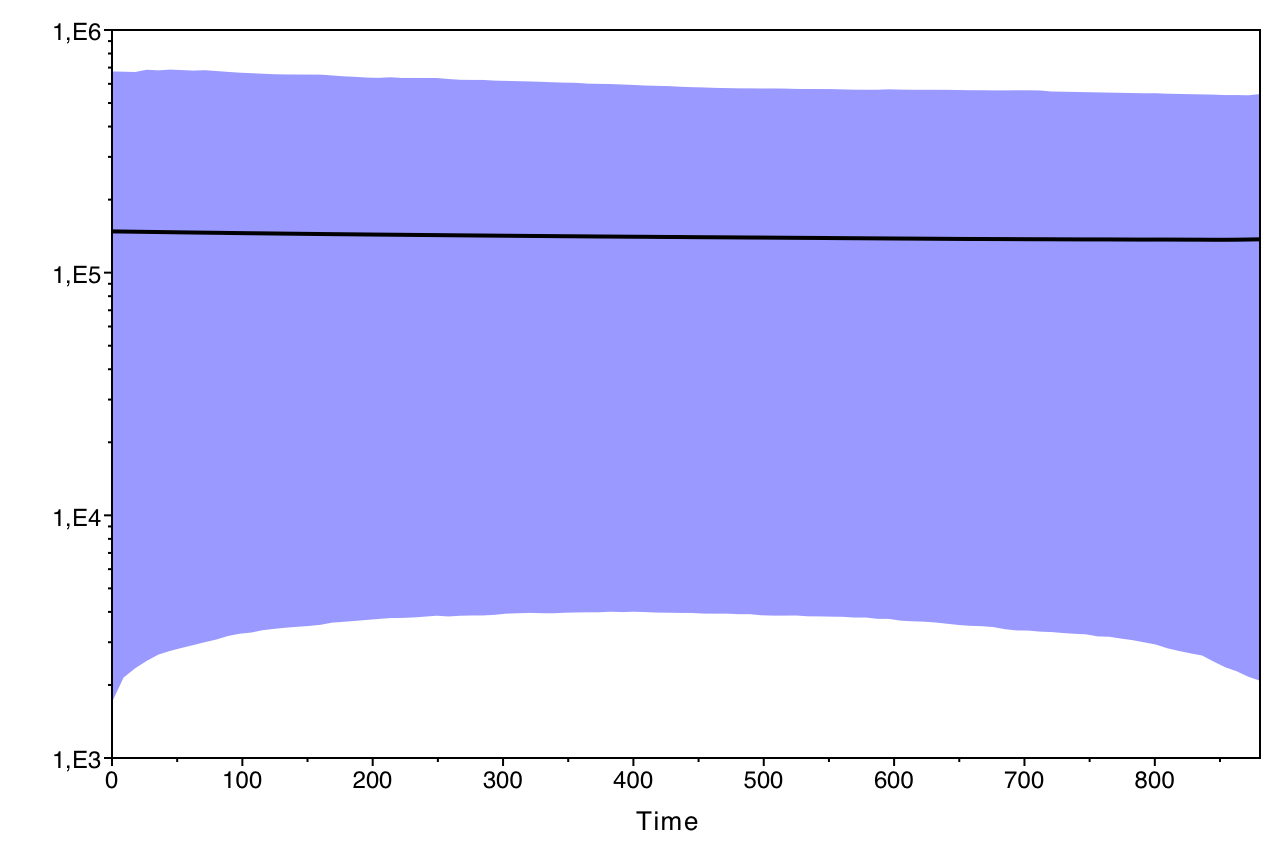
**

Supplement: Figure S3 — Bayesian Skyline Plot. The analyses were performed using the 49 Corsican sequences with date-tips for the medieval sequences (see text for details). X-axis: Time in years; Y-axis: Population size (Neτ) in log-scale. Mean is plotted with the 95% HPD. 3 runs of 100 M of iterations were performed (ESS >200 for all parameters). (DOC) [file pone.0030272.s003.doc]

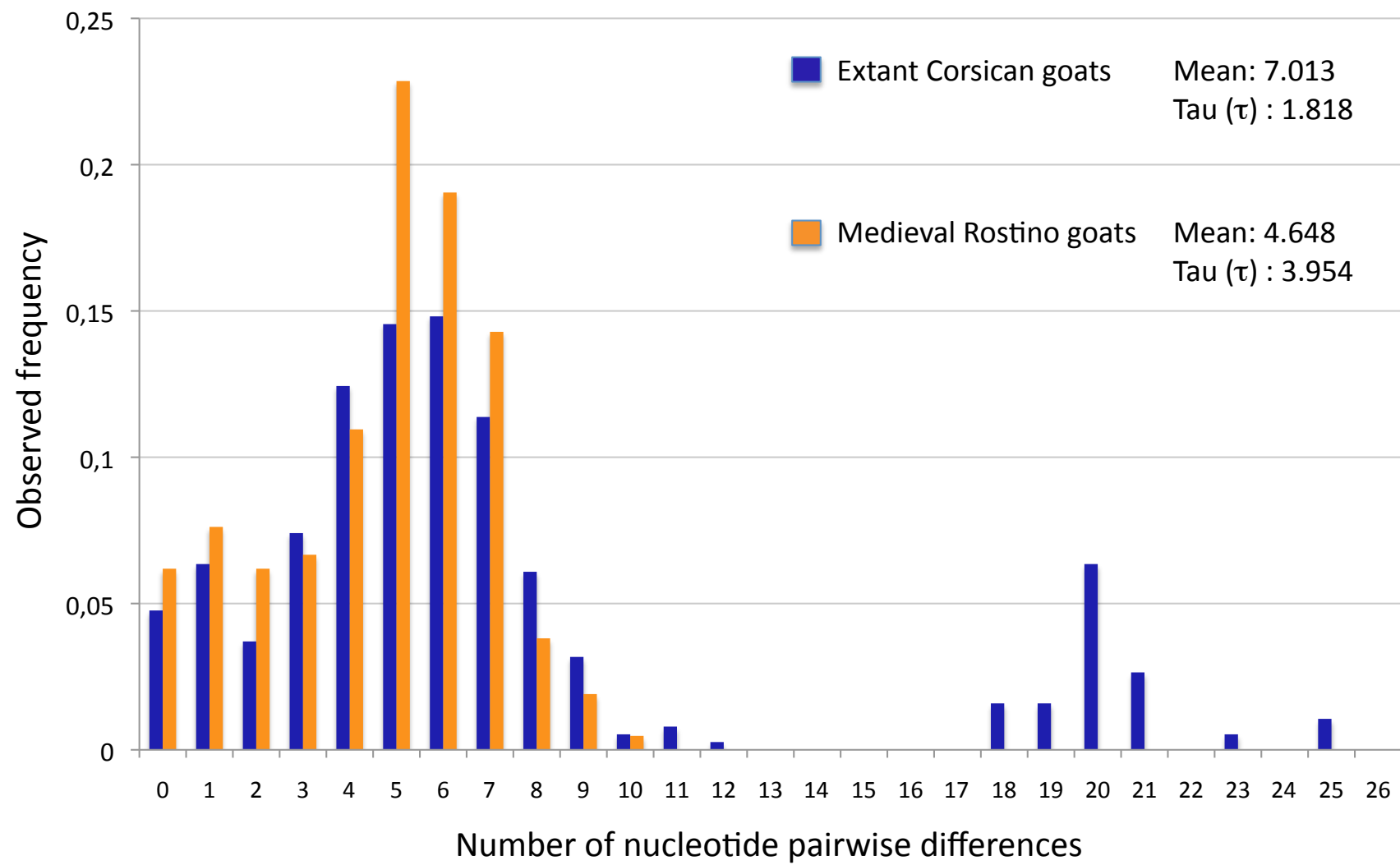

Supplement: Figure S4 — Mismatch distributions for medieval and extant Corsican goat populations. The numbers of pairwise differences are given on the x-axis and their frequency on the y-axis. (PDF) [file pone.0030272.s004.pdf]
