# Supplementary material for: A Dig into the Past Mitochondrial Diversity of Corsican Goats Reveals the Influence of Secular Herding Practices
Source: PLoS One. 2012 Jan 27;7(1):e30272. doi: 10.1371/journal.pone.0030272 (PMC3267719; doi:10.1371/journal.pone.0030272)
Supplement: Table S1 — Corsican goats sampling and mitochondrial genotyping results. A star indicates archeological samples for which the molecular identification was Ovis aries and a dash when no amplification was obtained. (DOC) [file pone.0030272.s005.doc]

| Medieval samples | Samples | Description | Locality/ period | Haplogroup | Haplotype | Accession number |
| --- | --- | --- | --- | --- | --- | --- |
|  | Ro-1 | Right frontal bone | Rostino  XII^th^ century | A | Ha 01 | JN007874 |
|  | Ro-2 | Right humerus |  | A | Ha 02 | JN007875 |
|  | Ro-3 | Left mandible |  | A | Ha 03 | JN007876 |
|  | Ro-4 | Right ulna |  | - | - | - |
|  | Ro-5 | Radius |  | A | Ha 04 | JN007877 |
|  | Ro-5 | Humerus |  | A | Ha 04 | JN007877 |
|  | Ro-5 | Tibia |  | A | Ha 04 | JN007877 |
|  | Ro-6 | Radius |  | A | Ha 05 | JN007878 |
|  | Ro-7 | Radius |  | A | Ha 04 | JN007879 |
|  | Ro-8 | Radius |  | A | Ha 06 | JN007880 |
|  | Ro-9 | Mandible |  | * | - | - |
|  | Ro-10 | Humerus |  | A | Ha 07 | JN007881 |
|  | Ro-10 | Humerus |  | A | Ha 07 | JN007881 |
|  | Ro-11 | Radius |  | A | Ha 08 | JN007882 |
|  | Ro-12 | Radius |  | A | Ha 09 | JN007883 |
|  | Ro-13 | Mandible |  | * | - | - |
|  | Ro-14 | Mandible |  | - | - | - |
|  | Ro-15 | Mandible | Rostino  XIV^th^ century | A | Ha 10 | JN007884 |
|  | Ro-16 | Mandible |  | A | Ha 02 | JN007885 |
|  | Ro-17 | Mandible |  | A | Ha 10 | JN007886 |
|  | Ro-18 | Mandible |  | A | Ha 11 | JN007887 |
|  | Ro-19 | Mandible |  | A | Ha 12 | JN007888 |
|  | Ro-20 | Mandible |  | A | Ha 04 | JN007889 |
|  | Ro-21 | Mandible |  | A | Ha 04 | JN007890 |
|  | Ro-22 | Mandible |  | A | Ha 09 | JN007891 |
|  | Ro-22 | Mandible |  | A | Ha 09 | JN007891 |
|  | Ro-23 | Mandible |  | A | Ha 13 | JN007892 |
|  | Ro-24 | Mandible |  | A | Ha 14 | JN007893 |
|  | Ro-25 | Mandible |  | A | Ha 04 | JN007894 |
| Present-day samples | CHFRCOR1 | | Altiani | A | Ha 04 | EF617738 |
|  | CHFRCOR2 | |  | A | Ha 04 | JN007895 |
|  | CHFRCOR3 | |  | A | Ha 15 | EF617755 |
|  | CHFRCOR4 | | Tralonca | C | Ha 26 | EF617787 |
|  | CHFRCOR5 | |  | A | Ha 04 | EF617739 |
|  | CHFRCOR6 | |  | C | Ha 26 | EF617786 |
|  | CHFRCOR7 | |  | A | Ha 16 | EF617756 |
|  | CHFRCOR8 | |  | A | Ha 17 | EF617757 |
|  | CHFRCOR9 | |  | A | Ha 09 | EF617758 |
|  | CHFRCOR10 | | Corte | A | Ha 18 | EF617759 |
|  | CHFRCOR11 | |  | A | Ha 15 | EF617760 |
|  | CHFRCOR12 | |  | A | Ha 19 | EF617761 |
|  | CHFRCOR13 | |  | A | Ha 06 | JN007896 |
|  | CHFRCOR14 | |  | A | Ha 20 | JN007897 |
|  | CHFRCOR15 | |  | A | Ha 19 | JN007898 |
|  | CHFRCOR16 | |  | A | Ha 09 | JN007899 |
|  | CHFRCOR17 | |  | A | Ha 17 | JN007900 |
|  | CHFRCOR18 | |  | A | Ha 09 | JN007901 |
|  | CHFRCOR28 | |  | A | Ha 07 | JN007906 |
|  | CHFRCOR29 | |  | A | Ha 25 | JN007907 |
|  | CHFRCOR19 | | Moltifao | A | Ha 21 | JN007902 |
|  | CHFRCOR20 | |  | A | Ha 04 | JN007903 |
|  | CHFRCOR22 | |  | A | Ha 09 | JN007904 |
|  | CHFRCOR23 | |  | A | Ha 22 | JN007905 |
|  | CHFRCOR25 | |  | A | Ha 23 | EF617740 |
|  | CHFRCOR26 | |  | A | Ha 07 | EF617741 |
|  | CHFRCOR27 | |  | A | Ha 24 | EF617742 |
|  | CHFRCOR31 | | Quenza | A | Ha 21 | JN007908 |
