# Supplementary material for: A Dig into the Past Mitochondrial Diversity of Corsican Goats Reveals the Influence of Secular Herding Practices
Source: PLoS One. 2012 Jan 27;7(1):e30272. doi: 10.1371/journal.pone.0030272 (PMC3267719; doi:10.1371/journal.pone.0030272)
Supplement: Table S2 — Population pairwise FST. Pairwise difference was used as distance method. FST values are given at upper right corner and corresponding p-values at bottom left. Significant p-values are highlighted in color (orange for intra-Corsica comparisons, pink for inter comparisons). NS: Non-significant, * p-value between 0.05 and 0.01, ** p-value between 0.01 and 0.001, *** p-value < to 0.001. (DOC) [file pone.0030272.s006.doc]

|  | XII | XIV | Medieval | Extant Corsican | Extant Corsican A only | All Corsican | All Corsican  A only | Sardinian | Portug. | Portug. A only |
| --- | --- | --- | --- | --- | --- | --- | --- | --- | --- | --- |
| XII |  | -0.02798 | -0.05824 | 0.04825 | 0.05105 | 0.00057 | -0.00119 | 0.07262 | 0.10259 | 0.10464 |
| XIV | NS |  | -0.05656 | -0.00623 | -0.01286 | -0.03598 | -0.03930 | 0.04846 | 0.04146 | 0.04271 |
| Medieval | NS | NS |  | 0.03609 | 0.02659 | -0.00677 | -0.01310 | 0.07018 | 0.07962 | 0.08095 |
| Extant Corsican | * | NS | * |  | -0.02227 | -0.01472 | 0.00330 | 0.05367 | 0.04497 | 0.04709 |
| Extant Corsican A only | * | NS | NS | NS |  | -0.01556 | -0.01632 | 0.04284 | 0.03120 | 0.03182 |
| All Corsican | NS | NS | NS | NS | NS |  | -0.01552 | 0.05076 | 0.04931 | 0.05066 |
| All Corsican  A only | NS | NS | NS | NS | NS | NS |  | 0.05241 | 0.04929 | 0.04991 |
| Sardinian | * | * | *** | *** | *** | *** | *** |  | 0.02206 | 0.02255 |
| Portuguese | *** | * | *** | *** | *** | *** | *** | *** |  | -0.00345 |
| Portuguese  A only | ** | * | *** | *** | * | *** | *** | *** | NS |  |
