# Supplementary material for: A Dig into the Past Mitochondrial Diversity of Corsican Goats Reveals the Influence of Secular Herding Practices
Source: PLoS One. 2012 Jan 27;7(1):e30272. doi: 10.1371/journal.pone.0030272 (PMC3267719; doi:10.1371/journal.pone.0030272)
Supplement: Table S3 — Genetic diversity parameters. Comparison for A and C haplogroups between Corsican and other Mediterranean or Portuguese datasets (see Figures S1, S3 and text). (DOC) [file pone.0030272.s007.doc]

|  |  | Number of sequences | HaC | Number of haplotypes | Haplotype diversity | Mean number of pairwise differences | Nucleotide diversity | Fs (Fu) | Tajima D |
| --- | --- | --- | --- | --- | --- | --- | --- | --- | --- |
| Corsican goats |  |  |  |  |  |  |  |  |  |
| All Corsican | A+C | 49 | 2 | 26 | 0.9473 ±0.0189 | 6.12 ± 2.96 | 0.0471 ± 0.0253 | -9.8130  (p: 0.0010) | -0.3022  (p: 0.4350) |
|  | A | 47 | - | 25 | 0.9473 ± 0.0189 | 6.12 ± 2.96 | 0.0471 ± 0.0253 | -9.8130  (p: 0.0010) | -0.3022  (p: 0.4350) |
| Other goats |  |  |  |  |  |  |  |  |  |
| Neolithic | A+C | 8 | 4 | 4 | - | - | - | - | - |
|  | A | 4 | - | 2 | - | - | - | - | - |
| Portuguese | A+C | 288 | 1 | 104 | 0.9790 ± 0.0030 | 5.30 ± 2.60 | 0.0414 ± 0.0221 | -24.8789  (p: 0.0000) | -0.7741  (p: 0.2560) |
|  | A | 287 | - | 103 | 0.9789 ± 0.0030 | 5.27 ± 2.56 | 0.0406 ± 0.0217 | -24.9047  (p: 0.0000) | -0.6702  (p: 0.3000) |
|  |  |  |  |  |  |  |  |  |  |
| North Mediterranean | A+C | 270 | 19 | 170 | 0.9899  ±0.0024 | 7.21  ±3.39 | 0.0555  ±0.0288 | -24.4726  (p: 0.0000) | -0.1529  (p: 0.5140) |
|  | A | 251 | - | 159 | 0.9887  ±0.0028 | 4.96  ±2.42 | 0.0381  ±0.0206 | -25.0930  (p: 0.0000) | -0.9013  (p: 0.1700) |
|  |  |  |  |  |  |  |  |  |  |
| Mediterranean Islands | A | 141 | 0 | 68 | 0.9832  ±0.0029 | 5.08  ±2.48 | 0.0391  ±0.0211 | -25.3315  (p: 0.0000) | -0.9526  (p: 0.1890) |
|  |  |  |  |  |  |  |  |  |  |
| South Mediterranean | A | 67 | 0 | 54 | 0.9914  ±0.0050 | 5.05  ±2.48 | 0.0389  ±0.0212 | -25.5288  (p: 0.0000) | -0.9380  (p: 0.1950) |
